# Mood Disorder Begins in the Mouth: Periodontitis Mediates Anxiety‐Like Behaviors

**DOI:** 10.1002/advs.76215

**Published:** 2026-06-19

**Authors:** Zhigang Chen, Xing Li

**Affiliations:** ^1^ College of Pharmacy Jinan University Guangzhou China; ^2^ State Key Laboratory of Natural Medicines China Pharmaceutical University Nanjing China

**Keywords:** anxiety, bone morphogenetic protein 4, C‐reactive protein, inflammation, periodontitis

## Abstract

The previous study elucidated that the C‐reactive protein (CRP)/bone morphogenetic protein 4 (BMP4) signaling pathway mediates periodontitis‐associated anxiety‐like behaviors in rats. This molecular crosstalk along the periodontal‐hippocampal axis provides novel insights into the etiology and mechanisms of emotional disorders. However, several key issues remain to be further discussed regarding the establishment of the core finding linking peripheral inflammation to emotional disorders and potential translational significance. Here, this commentary analyzes the mechanisms underlying CRP entry into the brain, the choice of behavioral paradigms for emotional assessment, the possible dynamic evolution of the pathogenesis, and the translational potential of CRP, with the aim of fostering constructive academic discussion.

Chronic periodontitis (PD) is a highly prevalent chronic inflammatory disease of the oral cavity worldwide. Epidemiological studies have shown a significant correlation between PD and various central nervous system (CNS) disorders, including anxiety and depression [1]. In this context, the oral‐brain axis challenges traditional understanding of the etiology of CNS diseases, which has historically been attributed to primary neuropathological changes within the brain. Nevertheless, several critical questions emerge, for instance, how local periodontal inflammation precisely affects distant emotional centers, such as the hippocampus, across anatomical barriers. A previous study has focused on the invasion of periodontal pathogens and the effects of inflammatory responses [2]. However, these detailed mechanisms can not fully account for the precise and specific regulation of brain function by oral inflammation, and a key bridge between the two remains lacking.

Recently, a study by Li et al. published in *Advanced Science*, provides a novel perspective for addressing the aforementioned questions [3]. This study ingeniously established a rat model of PD, revealing that PD alone is sufficient to induce anxiety‐like behaviors and impair hippocampal neurogenesis. Through a series of sophisticated techniques, including genetic techniques and single‐nucleus RNA sequencing (snRNA‐seq), the researchers delineated a significant finding regarding a signaling pathway from the periphery to the CNS. Specifically, PD elevates peripheral C‐reactive protein (CRP) levels, which then enter the hippocampus and regulate the expression of bone morphogenetic protein 4 (*Bmp4*) in oligodendrocyte progenitor cells (OPCs). This ultimately impairs hippocampal functions by inhibiting neuronal differentiation. This work not only identifies a CRP/BMP4 molecular axis linking oral diseases to mental disorders, but also highlights the key role of OPCs, long overlooked in neuroscience research in the mouth‐brain communication, thereby transcending the previous subjects that were confined to neurons, astrocytes, microglia, or their interactions.

In this study, Li et al. confirmed the rat PD model via behavioral and histomorphological assessments. Similar to the chronic restraint stress model, rats with ligature‐induced PD exhibited anxiety‐like behaviors, with immature neuron loss in the hippocampal dentate gyrus [3]. This evidence establishes the negative effects of PD on emotional homeostasis and hippocampal neurogenesis.

Next, to elucidate the molecular events linking PD and hippocampal damage, the researchers identified an obvious upregulation of CRP in the hippocampus of rats with PD, using an inflammatory antibody array. Interestingly, snRNA‐seq data showed no expression of *Crp* mRNA in various hippocampal cell types, while CRP levels were markedly increased in the peripheral blood and gingival tissues [3]. Thus, these vital findings suggest that the elevated CRP in the hippocampus mainly derives from the peripheral system. Notably, there are no significant changes in the blood‐brain barrier‐associated proteins ZO‐1 as well as CAV‐1 [3], and it was plausibly speculated that CRP may enter the CNS through unconfirmed mechanisms, such as CAV‐1‐independent transcytosis, or through conformational changes.

To further understand the functional necessity of CRP in this pathological process, Li et al. generated *Crp* knockout (KO) rats. In *Crp* KO rats, the PD‐induced anxiety‐like behaviors and hippocampal neurogenesis impairment were reversed [3], demonstrating CRP as an important hub bridging PD and central damage. Subsequently, *Bmp4* in the hippocampus was characterized as one of the most crucially downregulated genes through mRNA sequencing of *Crp* KO rats. In addition, overexpression of *Bmp4* in the hippocampus via adeno‐associated virus abolished the protective effects conferred by *Crp* KO [3], reproducing neurogenesis impairment as well as behavioral abnormalities, consequently indicating CRP as an upstream regulator of BMP4.

The researchers employed snRNA‐seq and determined where BMP4 originates and how it regulates neurogenesis. In detail, *Bmp4* in the hippocampus was primarily localized to OPCs, rather than in astrocytes or neurons [3], which have attracted considerable attention. In vitro cell experiments further confirmed that OPCs are the main producers of BMP4. Besides, *Bmp4* in OPCs of PD rats was upregulated, but this alteration was eliminated by *Crp* KO. Importantly, PD triggered the loss of OPCs in the hippocampus, whereas Crp KO rats, despite having fewer OPCs at baseline, maintained stable myelin basic protein levels under PD status [3], demonstrating greater functional resilience. Finally, Li et al. found that PD decreased the expression levels of multiple key genes involved in neuronal differentiation, including NeuroD1, but *Crp* KO reversed this phenomenon. Immunohistochemistry, combined with AAV‐*Bmp4* overexpression[3], collectively illustrated that CRP/BMP4 mediates the inhibition of neuronal differentiation.

The first point worth discussing in this study is the route by which CRP enters the brain. CRP exists in multiple conformations in vivo, as analyzed by the authors, and more specifically, circulating pentameric CRP (pCRP) can dissociate into monomeric CRP (mCRP). The latter exhibits greater pro‐inflammatory and tissue‐penetrating properties and disrupts endothelial intercellular junctions [4]. Although the present study found no changes in ZO‐1 or CAV‐1 expression [3], it remains possible that the inflammatory environment induced by PD drives the conversion of pCRP to mCRP, which then crosses the blood‐brain barrier through other mechanisms, such as downregulation or rearrangement of other tight junction proteins. Alternatively, other possibilities might exist, for example, there is a specific transporter or extracellular vesicle that mediates the transcellular transport of CRP. Elucidating these mechanisms would hold universal value for understanding how other peripheral inflammatory factors affect CNS function. Even for the impact of other similar peripheral diseases on the CNS, this still has referential value for theory and research paradigms. Collectively, this suggests that future studies should not regard the blood‐brain barrier merely as a static barrier, but should investigate its dynamic, selective permeability to different types of molecules under specific pathological conditions.

Additionally, in the elevated plus maze test, the time spent in the open arms, and in the open field test, the time spent in the center area are commonly used to assess levels of anxiety‐like behaviors in experimental animals [5], while the total distance traveled in the open field test is used to reflect spontaneous locomotion. These two behavioral paradigms used, however, are insufficient for capturing the degree of depression‐like behaviors in rats [3]. The inclusion of the tail suspension test, the forced swim test, and the sucrose preference test would better characterize depressive‐like phenotypes [5, 6], such as behavioral despair and anhedonia in rats. Animal models of PD exhibit a significant reduction in spontaneous locomotor activity during assessments of the open field test [3]. This raises a noteworthy concern that most behavioral paradigms, including the elevated plus maze test as well as the open field test, are closely associated with locomotion time or distance, and current data cannot rule out false positive results in behavioral tests caused by a direct effect of PD on motorium. Therefore, incorporating a rotarod test [7], could help improve the robustness of the conclusions.

Third, the PD model had a duration of 4 weeks. However, the effects of inflammation on the brain are dynamic, and chronic PD can persist for months or even years. It cannot be excluded that, as time progresses, BMP4 could be derived from OPCs and further interfere with astrocyte or microglial function, exacerbating neuroinflammatory responses and shifting the core cellular mediators through which PD influences neuropsychiatric diseases. Delineating the dynamic landscape of the oral‐brain axis pathology facilitates setting different intervention strategies in different time windows. In particular, while this study focused on the hippocampus, since the brain regions that regulate emotions function as complex networks, it remains to be determined whether PD exerts similar or distinct regulatory patterns in other emotion‐related regions, such as the ventral hippocampus, prefrontal cortex, and amygdala [8], under conditions of emotional disturbance, and whether these brain regions also show time‐dependent dynamics. Such regional specificity represents an important direction for future research.

Furthermore, CRP is a routinely measured clinical indicator, and alterations in its levels are associated with the risk of various diseases [9, 10]. Hence, whether it is safe to target CRP for intervention is still a question. After all, CRP is a component of the innate immune system, and the critical issue is whether inhibiting the function of CRP could lead to numerous adverse consequences. Based on real‐world translational considerations, more precise intervention strategies would greatly enhance feasibility. For example, developing drugs or novel delivery materials that specifically prevent CRP, mainly derived from peripheral circulation, from entering the specific functional brain regions, or that specifically block its signaling and function. Notably, the association of the evolutionary conservation of the CRP/BMP4 signaling axis in PD‐induced anxiety in humans has not been clarified.

In sum, the study by Li et al. not only provides a novel molecular explanation for how PD affects CNS function but also reveals a sophisticated regulatory pathway involving the interplay among the nervous system, the immune system, and the oral cavity. From a translational perspective, this research identifies CRP or BMP4 as potential intervention targets for neuropsychiatric complications associated with PD, representing a new direction for future drug development in emotional disorders, and offers a referable research paradigm.

## Author Contributions

**Zhigang Chen**: conceptualization, writing – original draft, writing – review and editing. **Xing Li**: writing – original draft.

## Conflicts of Interest

The authors declare no conflicts of interest.

## Data Availability

Data sharing not applicable to this article as no datasets were generated or analysed during the current study.
